# Feasibility of preservation of superior rectal artery plus dissection of lymph nodes around inferior mesenteric artery in laparoscopic resection for sigmoid colon cancer

**DOI:** 10.3389/fsurg.2023.1086868

**Published:** 2023-02-14

**Authors:** Haoyuan Ren, Yong Liu, Mingran Zhang, Liang An

**Affiliations:** Department of Gastrointestinal Surgery, The People's Hospital of Deyang, Deyang, China

**Keywords:** sigmoid colon cancer, superior rectal artery, inferior mesenteric artery, anastomotic leakage, overall survival

## Abstract

**Background:**

Limited data are available about superior rectal artery (SRA) preservation in laparoscopic resection for sigmoid colon cancer (SCC). This study aimed to evaluate the short-term and long-term efficacies of SRA preservation in laparoscopic radical resection for SCC.

**Methods:**

We retrospectively analyzed 207 patients with SCC who underwent laparoscopic radical resection for SCC from January 2017 to June 2021. A total of 84 patients received lymph node clearance around the inferior mesenteric artery (IMA) root (D3 lymph node dissection) with preservation of SRA (SRA preservation group), and 123 patients received high ligation of the IMA (control group). The clinicopathological data of the two groups were compared, and Kaplan–Meier method was performed to estimate patient survival.

**Results:**

Compared with the control group, the operation time of the SRA preservation group was longer (*p *< 0.001), but the postoperative exhaust and defecation times were significantly shorter (*p* = 0.003, *p *< 0.001). Two cases of postoperative ileus and four cases of anastomotic leakage were observed in the control group, whereas the SRA preservation group had none. However, no statistical difference was observed between the groups (*p *= 0.652, *p *= 0.248). The overall survival also showed no significant difference in (*p *= 0.436).

**Conclusion:**

Preservation of SRA plus dissection of lymph nodes around IMA did not increase postoperative morbidity and mortality nor affect the prognosis of patients but increased the bowel blood supply, which may have a significant positive effect on the recovery of postoperative intestinal function and reduction of anastomotic leakage.

## Introduction

For rectal cancer and sigmoid colon cancer (SCC), high ligation of inferior mesenteric artery (IMA) is performed for further lymph node dissection (1). High ligation of IMA with D3 lymph node dissection can improve the survival rate of patients (2, 3). However, insufficient perfusion of stumps after high ligation of IMA may lead to anastomotic ischemia (4–6). The blood supply of the proximal and distal stumps of anastomosis is an important factor affecting its healing. Preservation of superior rectal artery (SRA) theoretically increases the blood supply of the distal rectum and can reduce anastomotic leakage (AL) (7). However, reports on SRA preservation and its long-term efficacy are limited. In the present study, we investigated the short-term and long-term efficacies of SRA preservation to evaluate the clinical value of SRA preservation in SCC surgery.

## Methods

### Patient selection and data collection

In this retrospective cohort study, data were collected from patients who underwent laparoscopic radical resection of SCC at Department of Gastrointestinal Surgery, People’s Hospital of Deyang from January 2017 to June 2021.

The inclusion criteria were as follows: (1) complete clinicopathological data, (2) preoperative colonoscopy or intraoperative exploration of tumor located in sigmoid colon, and (3) adenocarcinoma confirmed by postoperative pathology.

The exclusion criteria included the following: (1) preoperative radiotherapy or chemotherapy, (2) tumor invading surrounding tissues and organs or distant metastasis, (3) intraoperative conversion to open surgery, (4) severe organ diseases, such as those of heart, lung, liver, and kidney, or hematopoietic and endocrine diseases that may affect survival, (5) complication with severe infection, intestinal obstruction, or other malignant tumors; (6) history of previous abdominal surgery.

**Figure 1:**
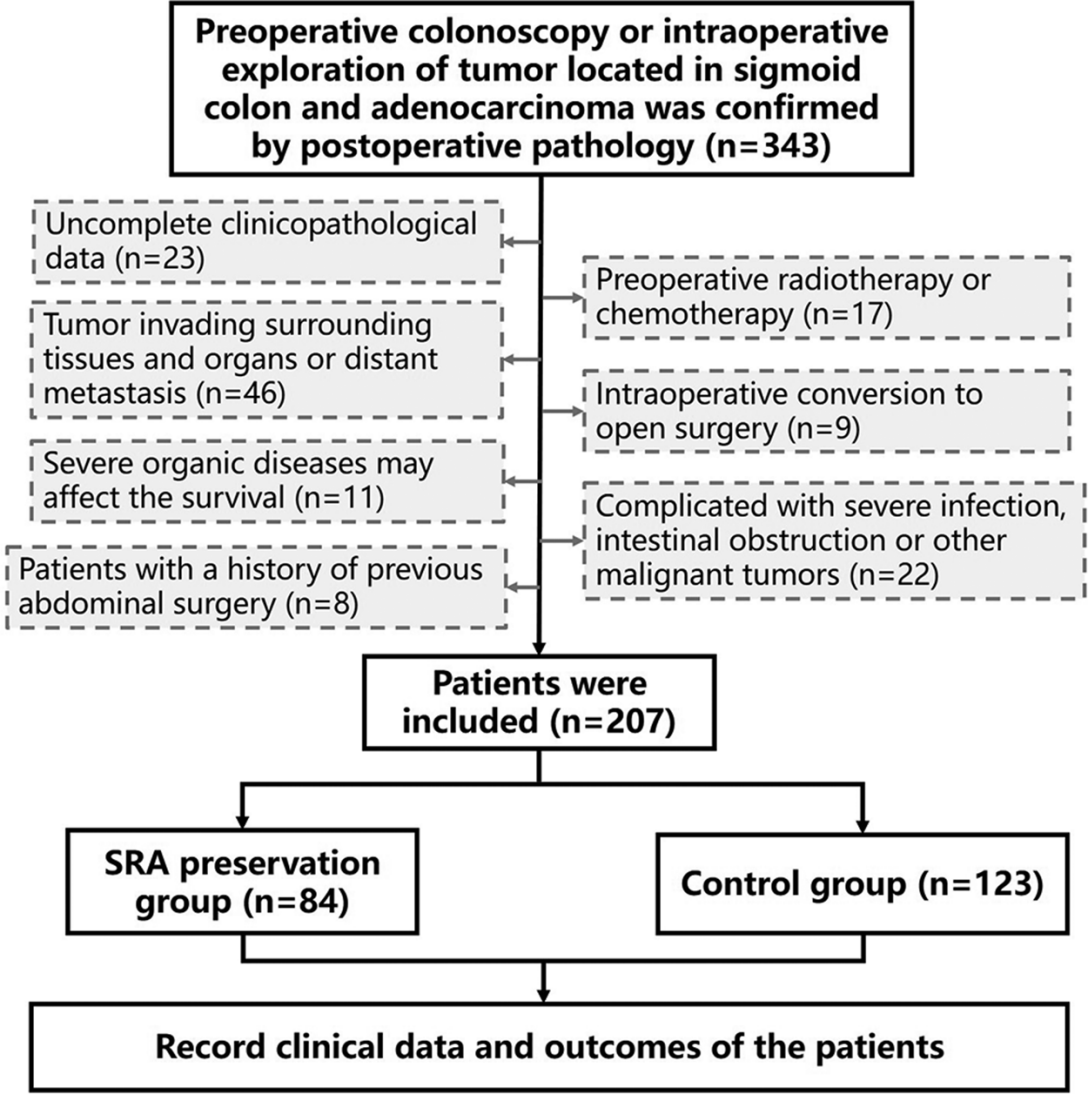
The flowchart of the study.

Based on SRA preservation, the patients were divided into the SRA preservation (*n* = 84) and control (*n* = 123) groups. The control group underwent conventional high ligation of the IMA root. All patient data, including age, gender, body mass index (BMI), operation time, intraoperative blood loss, tumor stage, the number of lymph nodes dissected, tumor differentiation, postoperative adjuvant chemotherapy, and postoperative exhaust and defecation times, were recorded in the database. Postoperative complications, mortality (30 days after surgery or before hospital discharge), and survival were also recorded (Figure 1).

### Surgical procedures

**Figure 2:**
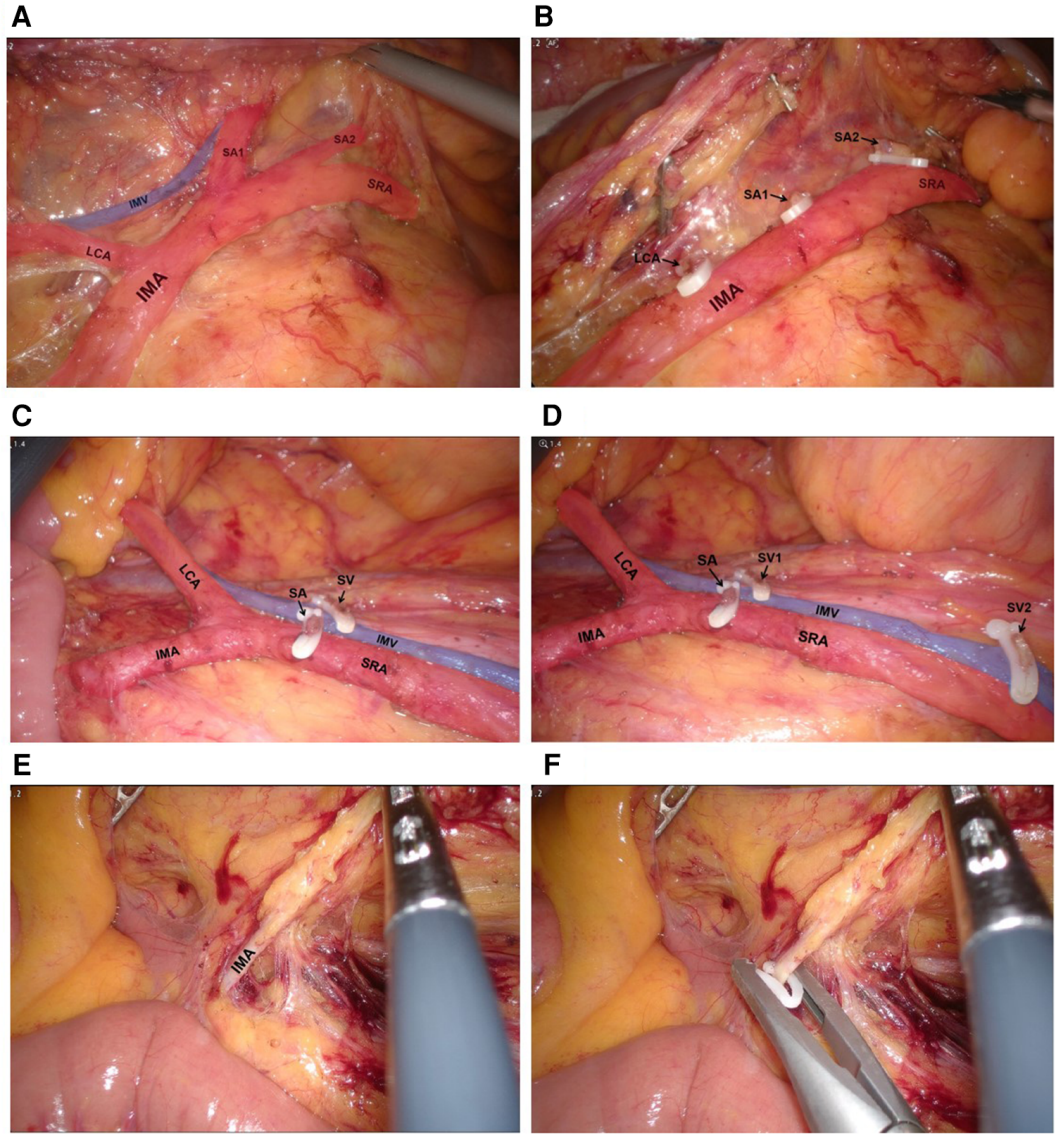
(**A,B**) Dissect the lymphatic adipose tissue around and at the root of IMA, divide and cut off LCA, SA1 and SA2. (**C,D**) Operative view of preserving LCA, IMV and SRA. (**E,F**) Operative view of high ligation of IMA.

Patients were laid in head-down lithotomy position. The peritoneal cavity was routinely explored. The peritoneum was opened on the right side of the IMA root, and the mesenteric membrane was separated upward along the space between Gerota's and Toldt's fascias. In the preservation group, the lymphoid adipose tissue at the root of IMA was dissected, and the IMA root was exposed. Then, the lymphatic adipose tissue around IMA was dissected along the direction of IMA, and the IMA vascular sheath was not opened routinely. The left colic artery and first or second sigmoid artery were divided and cut off along the IMA vascular sheath, and the SRA was preserved (Figures 2A,B). In addition to SRA, the left colic artery or inferior mesenteric vein (IMV) was selectively preserved in several patients, especially those with long sigmoid colon (Figures 2C,D). In the control conventional operation group, high ligation was performed at the roots of IMA and IMV roots (Figures 2E,F, respectively). Both groups followed the principle of complete mesocolic excision and 10 cm proximal and distal margin, and the inferior mesenteric plexus and hypogastric plexus were carefully protected. Before anastomosis, the bowels were fully freed to ensure that the anastomosis was free of tension.

### Postoperative complications

AL after colorectal resection is defined as the defect of intestinal wall integrity at the colorectal or colo-anal anastomosis site (including suture and staple lines of neorectal reservoirs), leading to a communication between intra- and extraluminal compartments. A pelvic abscess close to the anastomosis is also considered AL. The severity grading of AL is as follows: Grade A: AL requiring no active therapeutic intervention; Grade B: AL requiring active therapeutic intervention but manageable without re-laparotomy; Grade C: AL requiring re-laparotomy (8).

Postoperative ileus (POI) was diagnosed if two or more of the following criteria were met on or after postoperative day 4: absence of flatus for over 24 h, absence of stool for over 72 h, abdominal distension, nausea or vomiting, incapability to tolerate diet in the past 24 h; clinical and imaging examinations (including x-ray radiograph, sonography, and computed tomography) indicative of POI (9).

### Statistical analysis

Survival was measured from the time of surgery until death or last-known follow-up (censor date was January 1, 2022). The data were presented as mean ± standard deviation, and Chi-squared test or Fisher's exact test, analysis of variance, or group *t* test was conducted to test for differences between groups. Kaplan–Meier method was used for survival analysis, and log-rank test was applied for comparison of differences in survival curves. *p* < 0.05 was considered statistically significant. All statistical analyses were performed using the software SPSS Version 25.0.

### Ethical statement

The procedures of this study were in accordance with the Declaration of Helsinki and approved by the Ethics Committee of People's Hospital of Deyang (No.2022-04-051-K01).

## Results

### Comparison of clinicopathological factors and complications between patient groups

A total of 207 patients with SCC were enrolled in this study. Exactly 84 patients underwent D3 lymph node dissection with preservation of SRA (SRA preservation group), and 123 patients underwent high ligation of the IMA (control group). Table 1 shows the comparison of clinicopathological factors between SRA preservation and control groups. No significant differences were observed in gender, age, BMI, intraoperative blood loss, tumor stage, tumor differentiation, the number of lymph nodes dissected, and postoperative adjuvant chemotherapy (*p *> 0.05). However, given the anatomy of IMA branches and SRA preservation, the operation time was significantly longer in the preservation group (*p *< 0.001) (Tables 1, 2).

**Table 1:** Demographic and clinical characteristics.

	Control group (*n* = 123)	SRA preservation group (*n* = 84)	*F/χ* ^2^	*p*
Gender *n* (%)				
Male	76 (61.8)	56 (66.7)	0.51	0.473
Female	47 (38.2)	28 (33.3)		
Mean age (±S, years)	65.85 ± 11.87	64.64 ± 11.67	0.09	0.461
BMI (±S, Kg/m^2^)	21.51 ± 1.61	21.35 ± 1.37	2.158	0.47
Tumor stage *n* (%)				
I, II (%)	73 (59.3)	52 (61.9)	0.14	0.712
III (%)	50 (40.7)	32 (38.1)		
Tumor differentiation *n* (%)				
Poor (%)	9 (7.3)	11 (13.1)	1.91	0.167
Moderate, well (%)	114 (92.7)	73 (86.9)		
Postoperative adjuvant chemotherapy *n* (%)	55 (44.7)	41 (48.8)	0.34	0.562

**Table 2:** Operation data, postoperative complications and mortality.

	Control group (*n* = 123)	SRA preservation group (*n* = 84)	*F/χ* ^2^	*p*
Operation time (±S, min)	164.07 ± 22.04	175.66 ± 23.67	0.089	<0.001
Intraoperative blood loss (±S, ml)	48.74 ± 25.87	51.19 ± 27.07	0.972	0.512
Lymph node dissection (±S)	12.36 ± 8.84	11.44 ± 7.06	1.512	0.428
Postoperative exhaust time (±S, day)	2.04 ± 0.89	1.67 ± 0.86	1.482	0.003
Postoperative defecation time (±S, day)	3.11 ± 0.94	2.51 ± 0.78	0.008	<0.001
Morbidity (%)	6 (4.9)	0 (0.0)	2.66	0.103
Anastomotic leakage	4 (3.3)	0 (0.0)	1.33	0.248
Post-operative abdominal bleeding	0 (0.0)	0 (0.0)		NA
Lymphatic leakage	0 (0.0)	0 (0.0)		NA
Postoperative ileus	2 (1.6)	0 (0.0)	0.20	0.265
Reoperation (%)	0 (0.0)	0 (0.0)		NA
Mortality (%)	0 (0.0)	0 (0.0)		NA

**Table 3:** Univariate and multivariate survival analysis of prognostic factors for patients with SCC.

Factors	Univariate analysis			Multivariate analysis				
	*n*	*χ* ^2^	*p*	*β*	*SE*	Wald *χ*^2^	HR (95% CI)	*p*
Age		0.993	0.319					
≤59	64			-	-	-	-	
60	143							
Tumor differentiation		2.380	0.123	-	-	-	-	
Moderate, well	187							
Poor	20			0.471	0.459	1.052	1.602 (0.651–3.940)	0.305
Tumor stage		3.622	0.057					
I, II	124			-	-	-	-	
III	83			1.442	0.596	5.859	4.228 (1.316–13.586)	0.015
Lymph node metastasis		0.04	0.834					
No	125			-	-	-	-	
Yes	82			−0.829	0.606	1.871	0.436 (0.133–1.432)	0.171
Adjuvant chemotherapy		32.410	<0.001					
No	111			-	-	-	-	
Yes	96			−1.360	0.392	12.044	0.257 (0.119–0.553)	0.001
SRA preservation		0.606	0.436					
No	123			-	-	-	-	
Yes	84			0.510	0.336	2.303	1.665 (0.862–3.218)	0.129

The postoperative exhaust and defecation times of the SRA preservation group were significantly shorter than those of the control group, and the differences were statistically significant (*p *= 0.003; *p *< 0.001) (Table 2). Two cases of POI were observed in the control group, but the difference was not statistically significant compared with that in the preservation group (*p *= 0.652). Four cases of AL were detected in the control group and none in the preservation group, but the difference was not statistically significant (*p *= 0.248). No postoperative abdominal bleeding, lymph leakage, death, nor reoperation occurred in the two groups after surgery (Table 2).

### Survival

**Figure 3:**
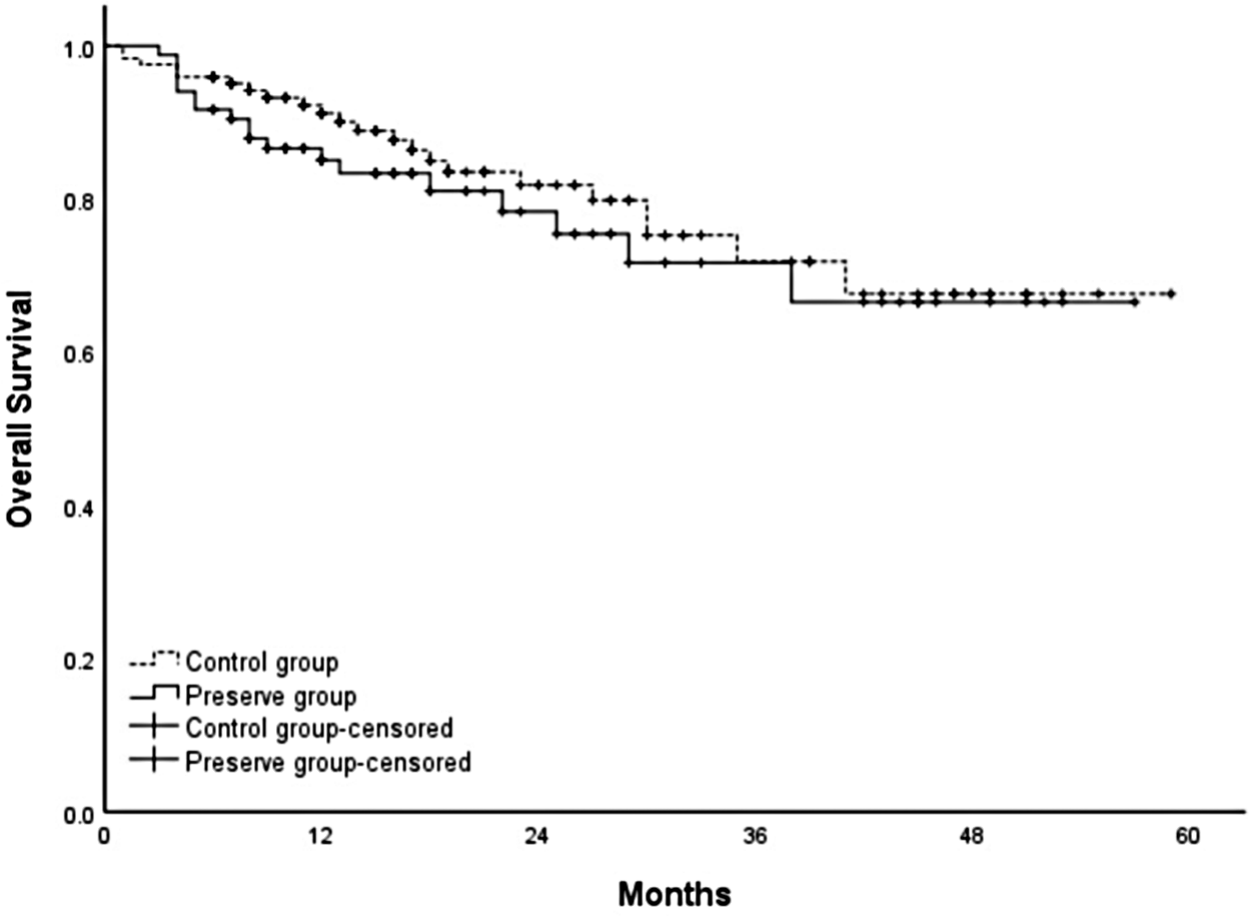
Comparison of overall survival between SRA preservation group and control group (*p *= 0.436).

**Figure 4:**
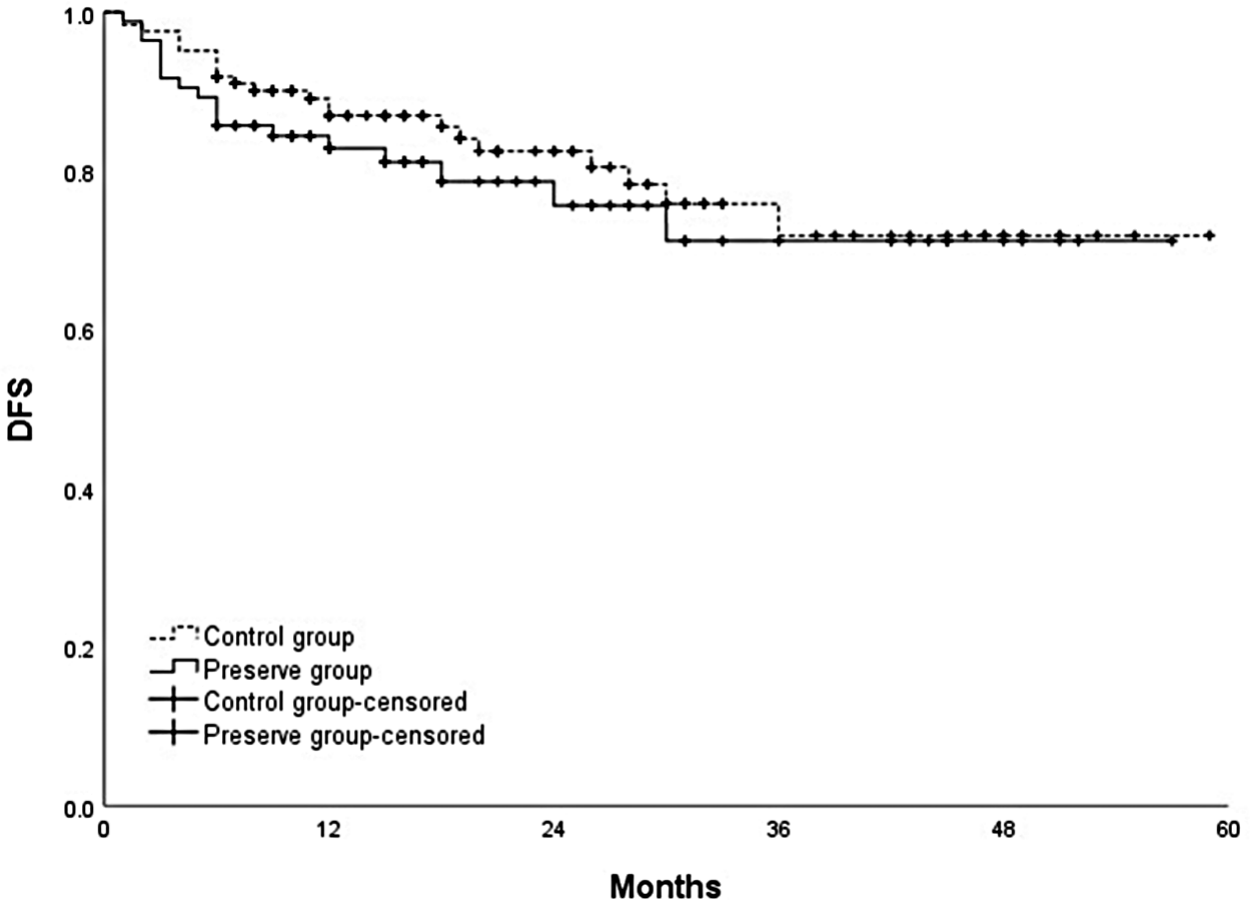
Comparison of disease-free survival between SRA preservation group and control group (*p *= 0.414).

The cumulative 1-, 2-, 3-, and 4-year overall survival rates were 88.7%, 80.4%, 71.7%, and 67.3%, respectively. No significant difference was observed in the overall survival and disease-free survival between the two groups (*p *= 0.436; *p *= 0.414) (Figures 3, 4, respectively).

Univariate analysis revealed that adjuvant chemotherapy (*p* < 0.001) was significantly associated with overall survival. Factors that may affect patient survival were further included in multivariate analysis through the Cox proportional risk model. Multivariate analysis demonstrated that tumor stage (*p* = 0.015) and adjuvant chemotherapy (*p *= 0.001) remained independently associated with overall survival. Adjuvant chemotherapy was a protective factor affecting the survival of patients [hazard ratio (*HR*) = 0.257, 95% confidence interval (*CI*) (0.119, 0.553, *p *= 0.001], and tumor stage was an adverse factor [*HR *= 4.2278, 95% *CI* (1.316, 13.586), *p *= 0.015]. However, SRA preservation was not an independent prognostic factor in this cohort (Table 3).

## Discussion

High ligation of the IMA is usually recommended during sigmoid colon and rectal cancer surgery to achieve complete D3 lymph node dissection. Several surgeons indicated that high ligation of the IMA for colorectal cancers can improve lymph node harvest, which facilitates accurate tumor staging and thus better disease prognostication, and may improve the survival rate of patients (10, 11). However, an increasing number of studies or meta-analyses showed no significant difference in the number of lymph nodes dissected and overall survival between IMA high ligation versus IMA low ligation (12–15). Similarly, our study compared IMA high ligation with SRA preservation during SCC surgery. No difference was observed in the number of lymph nodes removed or overall survival between the two procedures. The results indicated that SRA preservation during SCC surgery does not increase postoperative morbidity and mortality, does not affect prognosis, and can achieve D3 lymph node dissection, which is technically completely feasible.

However, most previous studies showed that IMA high ligation was associated with a high incidence of AL (14, 15). Early AL is mostly related to technical failure of the anastomosis due to iatrogenic surgical disruption of the peri-anastomotic microvascular blood supply or tension at the anastomotic site (16), whereas late AL is related to pre-existing conditions, such as local sepsis, poor nutrition, immunosuppression, obesity, vascular disease, and radiation exposure (17). A recent study performed real-time indocyanine green fluorescence angiography to measure fluorescence time as a marker of blood flow in the proximal and distal stumps before anastomosis. The results showed that IMA high ligation significantly prolonged fluorescence time in the sigmoid colon during anterior rectal resection compared with low ligation, which indicates that IMA high ligation induced significant colon stump hypoperfusion (4). This finding possibly explains why IMA high ligation resulted in a high rate of AL. As a result, an increasing number of surgeons are opting to perform low ligation of the IMA with D3 lymph node dissection (18–23), particularly in patients with diabetes, obesity, or old patients with cerebrovascular disease and atherosclerotic vessels. Similarly, for SCC, SRA preservation can theoretically increase the blood supply to the distal stump of sigmoid colon or upper rectum and reduce the incidence of AL. However, reports on the preservation of SRA procedure are limited. Our study showed that AL occurred in four cases in the control group but not in the preservation group. Although no statistical difference was observed between the two groups, no AL occurred when SRA was preserved. Whether the differences will become more pronounced if the sample size is large enough should be determined. These results indicate that SRA preservation may have a certain positive significance in reducing the incidence of AL.

In addition, our study revealed that the postoperative exhaust and defecation times were significantly shortened after SRA preservation, and no POI was observed. In preservation of SRA, increased bowel blood supply has significant positive effects on postoperative intestinal function recovery, which is conducive to enhanced recovery after surgery.

## Conclusion

In conclusion, SRA preservation plus dissection of lymph nodes around IMA in laparoscopic radical resection for SCC did not increase postoperative morbidity and mortality and did not affect patient prognosis. However, this process may increase the bowel blood supply, which may have a significant positive effect on the recovery of postoperative intestinal function and reduction of AL. Without compromising oncological outcomes, this alternative surgical technique should be recommended as a better intraoperative treatment option for reducing postoperative complications. However, certain technical difficulties need to be addressed by experienced surgeons under the premise of technical assurance. Large and well-designed multicenter randomized clinical trials are necessary to confirm these findings and shed light on this topic.

## Data Availability

The original contributions presented in the study are included in the article/Supplementary Material, further inquiries can be directed to the corresponding authors.
